# Respectful maternity care and its related factors in maternal units of public and private hospitals in Tabriz: a sequential explanatory mixed method study protocol

**DOI:** 10.1186/s12978-020-0863-x

**Published:** 2020-01-20

**Authors:** Khadije Hajizadeh, Maryam Vaezi, Shahla Meedya, Sakineh Mohammad Alizadeh Charandabi, Mojgan Mirghafourvand

**Affiliations:** 1 Student of Midwifery, Students’ Research Committee, Midwifery Department, Tabriz University of Medical sciences, Tabriz, Iran; 2 Fellowship of gynecology oncology, Alzahra teaching hospital, Tabriz University of Medical Sciences, Tabriz, Iran; 3 Member of South Asia Infant Feeding Research Network (SAIFRN), School of Nursing, Faculty of Science, Medicine and Health, University of Wollongong, Wollongong, Australia; 4 Department of Midwifery, Faculty of Nursing and Midwifery, Tabriz University of Medical Sciences, Tabriz, Iran; 5 Social Determinants of Health Research Center, Tabriz University of Medical Sciences, Tabriz, Iran

**Keywords:** Disrespect, Abuse, Respect, Childbirth, Mixed method

## Abstract

**Background:**

Disrespect and abuse (D&A) can violate human rights, affect women’s decisions on the type of delivery method, and exacerbate their mental health conditions; therefore, this study aims to: a) assess the status of D&A and respectful maternity care (RMC) during childbirth and their relationships with childbirth experience, socio-demographic and obstetrics characteristics; b) explain women’s perceptions of various RMC aspects and determinants during childbirth; and c) present a guideline for promoting of RMC.

**Methods/design:**

A mixed methods sequential explanatory design will be used to conduct this study in 3 phases. The first phase is a quantitative study with a longitudinal descriptive-analytical design to identify any D&A and RMC and their relationships with childbirth experience among 334 women who have given birth in public and private hospitals in Tabriz, Iran. The sample will be selected proportional to each population. The second phase is a qualitative study to explore women’s perceptions of various RMC aspects and their determinants during childbirth. The conventional content analysis approach will be used to analyze the data. The third phase is focused on developing a guideline to improve the quality of maternity care. The literature review, findings of phase one and two, and focus group discussion (FGDs) with staff in the labour ward and using a Delphi technique will be used to complete the final phase.

**Discussion:**

Considering the vulnerability of women during labor and delivery and the effect of D&A on cesarean section rates, a supportive guideline can improve the quality of maternity care and reduce D&A during childbirth, and improve women’s childbirth experiences.

**Ethical code:**

IR.TBZMED.REC.1398.202.

## Plain English summary

Disrespect and abuse (D&A) is a global phenomenon that affect women’s decisions on the type of delivery method, lactation, childbirth experience and mental health condition. The first phase is a quantitative study with a longitudinal descriptive-analytical design to identify any D&A and RMC and their relationships with childbirth among women who have given birth at public and private hospitals in Tabriz. The second phase is a qualitative study to explore women’s perceptions of various RMC aspects and their determinants during childbirth. The findings of the qualitative and quantitative study in addition to the literature review and focus group discussion (FGDs) will be used to developing a guideline. The increasing growth of cesarean section rates highlights the urge to develop a supporting guideline to improve the quality of maternity care, reduce D&A during childbirth, and improve women’s childbirth experiences.

## Background

The World Health Organization (WHO) has issued a statement on disrespect and abuse (D&A) during childbirth, which emphasized the importance of respectful maternity care (RMC) and women’s rights during pregnancy and childbirth, and the need for immediate attention to this global phenomenon [1]. D&A has been recognized as an important issue since 1950; however, it was developed when human rights organizations began collecting evidence on D&A in 2007 [2–4]. Bowser and Hill (2010) conducted an analysis with a global perspective and presented 7 categories for D&A including physical abuse, discrimination, non-consented clinical care, non-dignified care, non-confidential care, abandonment of care, and detention in health facilities [5]. D&A during labor and childbirth have increased over the past decade [6]. Early studies have reported different prevalence rates ranging from 20% in Kenya [7] to 98% in Nigeria [8]. The review of relevant articles shows that some multifactorial causes including lack of professional support for health care staff, hierarchical work relations, excessive workload, inadequate staff at different levels, and poor infrastructures can contribute to the increased prevalence of D&A [5, 9].

Evidence suggests that women may be unwilling to have normal-vaginal delivery (NVD), if they experience D&A during maternity care [10, 11]. In a review article in Iran, Azami et al. (2014) found that in nearly 40% of the cases, mothers selected cesarean section because of their fear of NVD [12]. There is also evidence that mothers may not seek maternity care, if they face D&A [13]. The WHO emphasized the important role of RMC in statements entitled “RMC improves lactation” [14] and “recommendations for improving the childbirth experience” [15]. D&A can violate human rights and exacerbate women’s mental health conditions such as sleep disorders and post-traumatic stress disorder (PTSD) [16].

Despite the abundance of research on D&A prevalence, few studies have investigated effective measures for reducing and preventing the prevalence of D&A behaviors during labor and birth [17–19]. The identification of both aggravating and mitigating factors of negative and abusive care provider-patient relationships has been neglected in health systems. Several countries have studied the role of D&A in childbirth and its related factors. Quantitative studies conducted in other countries to determine the level and type of D&A show high abuse rates, especially in African countries [8, 20]. Few studies were found to assess women’s D&A experiences during childbirth [21–24]. In Iran, no rigorous study was found to use a standard measurement tool to assess and manage RMC. However, given the importance of the cultural, economic, and social differences among various societies, this issue must be investigated inclusively. A mixed methods research can provide the best approach to identify the indicators of D&A while women’s experience is explored and considered in developing new guidelines The mixed methods approach emphasizes epistemological pluralism; hence, it supports the integration of different and even contradictory theories, approaches and methods, it help researchers better understand various concepts [25].

Regarding the importance of labor and birth, and the impact of D&A on women’s decisions on the type of birth method [10, 11], it is necessary to assess the status of D&A and its related factors to improve RMC behaviors.

### Study aim

This study is aimed to assess the status of D&A and RMC during childbirth, their impact on women’s experience (quantitative phase) and explore women’s perceptions of RMC aspects during childbirth (qualitative phase) to develop a new guideline to improve maternity care in an Iranian population.

### Specific objectives

1. Determining psychometric properties (including face validity, content validity, construct validity, reliability, repeatability, and internal consistency) of RMC and D&A scales.

2. Determining central tendency and dispersion indices for RMC scores in pregnant women who have given birth at public and private hospitals in Tabriz, Iran.

3. Determining the frequency of D&A in pregnant women who have given birth at public and private hospitals in Tabriz, Iran.

4. Determining central tendency and dispersion indices for childbirth experience scores in pregnant women who have given birth at public and private hospitals in Tabriz, Iran.

5. Determining the relationship between D&A and childbirth experience in pregnant women who have given birth at public and private hospitals in Tabriz, Iran.

6. Determining the relationship between RMC and childbirth experience in pregnant women who have given birth at public and private hospitals in Tabriz, Iran.

7. Determining the relationship between socio-demographic and obstetrics characteristics with D&A and RMC in pregnant women who have given birth at public and private hospitals in Tabriz, Iran.

8. Exploring women’s perceptions of various RMC aspects and determinants during childbirth in pregnant women who have given birth at public and private hospitals in Tabriz, Iran.

9. Developing a RMC guideline at public and private hospitals in Tabriz, Iran.

## Methods/design

### Study design

**Fig. 1:**
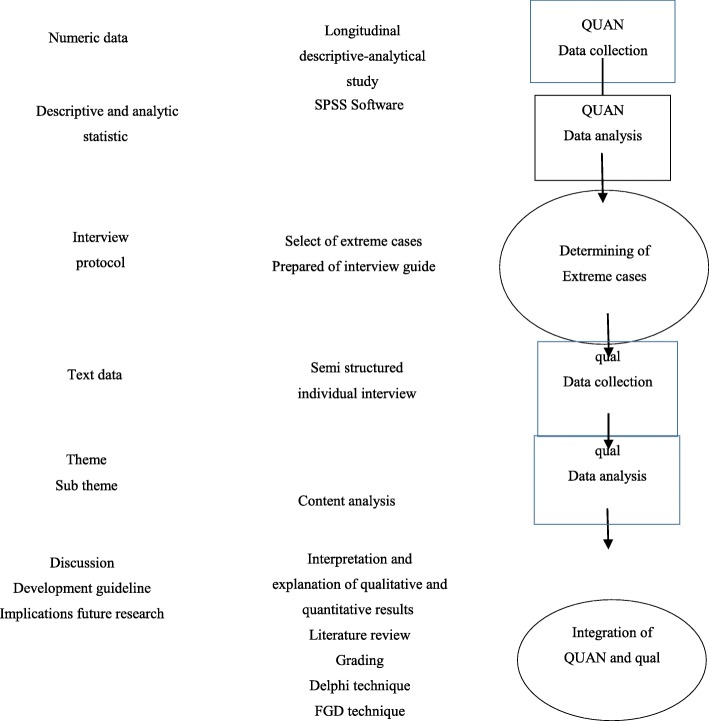
Study diagram

A mixed methods sequential explanatory design will be used to conduct this study by collecting, analyzing, and integrating the quantitative and qualitative data. Mixed methods are based on the principles of pragmatism paradigm, based on which, the integration of quantitative and qualitative approaches enhances our understanding of an issue or problem. In this study, the quantitative and quantitative data will be collected in the first and second phases, respectively. The qualitative data will be expanded and explained the collected quantitative data in the first phase, and both the quantitative and qualitative data will be integrated in the discussion section and will used to develop the guideline [26, 27] (Fig. 1).

### Phase one: quantitative study

Phase one is a longitudinal descriptive-analytical study that will be carried out to assess D&A and RMC 6–18 h after birth and then childbirth experience will be assessed after 30–45 days. The RMC and D&A scales will be validated prior to data collection. Then, the relationship between D&A and RMC with childbirth experience and socio-demographic and obstetrics characteristics will be investigated in a group of Iranian women. The target population includes women who have given birth at public and private hospitals in Tabriz, Iran.

### Sample size and sampling method

To validate RMC and D&A scales in Farsi language, 230 women will be recruited to unitize the factor analysis. Nunnally and Bernstien suggest selecting 10 participants per item in factor analysis. The D&A scale contains 23 items; therefore, 230 participants will be needed [28]. Considering a follow-up attrition rate of 20%, this number will be increased to 275. Based on a study in Ethiopia [29], and considering precision (d) = 0.05, Z = 1.96, *p* = 0.32 (any mistreatment of women, at least one form of mistreatment of women in hospitals), the sample size for the quantitative part of the study was calculated as 334.

### Sampling

Following the approval of the research project by the Ethics Committee of Tabriz University of Medical Sciences, the study will be conducted on 334 women who have given birth at public (Al-Zahra, Taleghani) and private (Shams, 29 Bahman, Behboud, Noor-e Nejat, Shahriar) hospitals in Tabriz. The researchers will visit the hospitals, identify eligible women by using the hospital birth statistic system, inform them about the research objectives and method, and obtain their informed consent if they are willing to participate in the study. The participants will complete the socio-demographic and obstetrics characteristics questionnaire, and the RMC and D&A scales 6–18 h after the childbirth. The childbirth experience questionnaire (CEQ2) will be completed 30–45 days after childbirth.

### Inclusion criteria

The women who had vaginal birth and live in Tabriz will be invited to participate in the study.

### Exclusion criteria

The exclusion criteria are: taking antidepressants over the past year, experiencing stressful incidents such as divorce, facing the death of a family member or diagnosis of a family member with an incurable or life-threatening disease within the past 3 months, suffering from a mental disability, being deaf and mentally disabled, still birth and giving birth to a baby with a major anomaly.

### Scales and data collection

The socio-demographic and obstetrics characteristics questionnaire, the RMC and D&A scales, and the CEQ2 will be used to collect the quantitative data. Face-to-face interviews will be conducted to collect the data. The socio-demographic and obstetrics characteristics questionnaire includes questions about age, educational qualifications, occupation, having a companion, religion, ethnicity, marital status, residential status, family income, living place (city or village), type of hospital (private or public), number of pregnancies, number of deliveries and abortions, history of stillbirth and infertility, drug use, history of sexually transmitted diseases, quality of prenatal care, interventions in childbirth (via drug, etc.) by the midwife or the gynecologist, patients’ freedom and comfort during labor and delivery, length of labor, participation in physiologic childbirth classes, length of stay in the delivery room, number of maternity care providers during the delivery, various delivery complications, delivery time (day or night), previous delivery place, delivery staff (physician, midwife), and gestational age at delivery.

The RMC scale has four domains and 15 items including friendly care (7 items), abuse-free care (3 items), timely care (3 items), and discrimination-free care (2 items). The responses are scored using the following pattern: “totally agree” (score 5), “agree” (score 4), “I do not know or indifference” (score 3), “disagree” (score 2)”, and “totally disagree” (score 1). Items with negative meanings are scored using negative digits. This tool will be completed 6–18 h after the childbirth. The validity and reliability of this tool (α = 0.845) were approved by Sheferaw (2016) in Ethiopia [30]. Higher means indicate more positive childbirth experience of RMC.

The D&A scale consists of 7 statements and 23 items including protecting women from physical harm (6 items), protecting their rights for getting information about their condition/informed consent/their preferences (8 items), maintaining their confidentiality and privacy (1 item), maintaining their dignity and respect (2 items), providing them with equitable and discrimination-free care (2 items), taking care of pregnant women (never left without care/ attention) (3 items), and discharging them upon their request (never detaining or confining them against their will) (1 item). The score zero (if the response is no) or one (if the response is yes) is given to each item to measure the frequency of D&A for each item. If any of the items in a statement are positive, then a D&A will be considered for that statement. This scale was designed by Asefa (2015) [31] and approved by Maternal and Child Health Integrated Program (MCHIP) [32].

The Childbirth Experience Questionnaire-2 (CEQ2) consists of 22 items, which measures women’s childbirth experience. The questionnaire also consists of four domains: own capacity (sense of control, personal feeling about childbirth and labor pain), professional support (midwifery information and care), perceived safety (feeling of safety and positive memories from childbirth), and participation (ability to change position, move, and have a say in the choice of pain relief). A 4-point Likert Scale is used to score 19 items, and the other three items are assessed using a visual analogue scale (VAS). The validity and reliability of the tool has been approved by the American Association of Women. The responses are scored using the following pattern: “totally agree” (score 1), “partly agree” (score 2), “partly disagree” (score 3), and “totally disagree” (score 4). The visual scale values will be converted to scores from 1 to 4: 0–40 (score 1), 41–60 (score 2), 61–80 (score 3), and 80–100 (score 4). Items with negative meanings are scored using negative digits. Higher CEQ2 scores indicate a more positive childbirth experience. This tool will be completed 30–45 days after childbirth [33]. The psychometric properties of the tool have been approved by Mirghafourvand et al. in a PhD project.

The validity of socio-demographic and obstetrics characteristics questionnaire will be measured using content and face validity. The content, face and construct validity of the Farsi version of RMC and D&A scales will be measured following a forward and backward translation process. In a study on a pilot sample of 20 individuals, the two-week test-retest reliability of RMC and D&A scales will be measured, and the Cronbach’s alpha coefficient (internal consistency index) and Intra-class Correlation Coefficient (ICC) will be determined.

### Data analysis

The quantitative data will be analyzed with SPSS-24. Socio-demographic and obstetrics characteristics and RMC and D&A scales will be described by frequency (percent), as well as mean (standard deviation) if the data are normally distributed or median (25 to 75 percentile) if they are not normally distributed. To determine the relationship between RMC and D&A and childbirth experience, bivariate analyses (including independent-t and Pearson correlation tests) will be conducted, which are followed by a multivariate analysis (multivariable linear regression test) to control the effect of confounding variables. To determine the relationship between socio-demographic and obstetrics characteristics with RMC and D&A, bivariate analyses (including Pearson correlation test, chi-square, independent *t*-test and one way ANOVA) will be conducted. Then, a backward multiple regression analysis will be conducted to control the effect of confounding variables.

### Phase two: qualitative study

The second phase consists of an exploratory qualitative study with content analysis. This qualitative method will be used to explore and explain women’s perceptions of various RMC aspects and determinants during childbirth.

### Sampling method

Purposive sampling will be used to explore women’s perceptions of RMC aspects and determinants during childbirth. The extreme cases on the two sides of the overall RMC score spectrum from phase one (the uppermost and lowermost 10% extreme values) will be selected as the participants in the qualitative phase of the study. A conventional qualitative content analysis approach will be adopted in this study. The main advantage of this approach is obtaining direct information from a study without imposing preconceived categories or theories. Following the completion of the quantitative sampling, the completed RMC questionnaire will be analyzed, extreme cases will be determined, and interviews will be carried out. In addition, participants with specific differences (in some variables) as well as those for whom unexpected results are found will be interviewed.

### Data collection

The qualitative data will be audio-recorded during semi-structured in-depth interviews with open-ended questions. During interviews, the researchers will encourage participants to express their views and experiences freely [34]. Prior to the qualitative stage, the interview guide will be developed by designing questions based on the findings of the quantitative study and related factors. To obtain credible data, the research team members will begin the interviews with predesigned questions, analyze responses to each question, and raise in-depth and exploratory questions such as “What do you mean?” “Why?” “Please explain further,” and “Please give an example.” The participants will select the location where they are willing to be interviewed. The sampling will continue until data are saturated [34].

### Data analysis

A conventional qualitative content analysis approach will be adopted to analyze the data. In this approach, the researchers read and interpret all available texts to get a complete understanding of them. Then, the texts are read word-for-word to extract relevant codes. The main advantage of this approach is obtaining direct information from a study without imposing preconceived categories or theories [35]. The data will be analyzed based on a qualitative content analysis method introduced by Graneheim and Lundman [36]. This method allows for extracting not only the explicit content of the texts, but also their implicit content with varying degrees of abstraction. We will use four criteria to evaluate the accuracy of the qualitative data (Credibility, Dependability, Confirmability, Transferability) [37]. The interview texts and codes were organized in MAXQDA.

### Phase three: developing a guideline to improve maternity care

The integration of the quantitative and qualitative results can help the researchers develop a guideline for improving the quality of maternity care provided at maternity wards. However, there will be a systematic review of literature to \identify the best practice. The following key terms will be used to search PubMed, Scopus, Embase, Google scholar and Cochrane library. Then, the quality of the obtained data will be evaluated using Grading of Recommendations, Assessment, Development and Evaluation (GRADE) GRADE approach, the evidence and data will be analyzed, to develop the primary version of the guideline. The final version of the guideline will be developed by Delphi technique and focus groups among labor and delivery staff including midwives, obstetric team, manager, educators and other health care providers.

## Discussion

The increasing growth of cesarean section rates, highlights the urge to develop a supporting guideline to improve the quality of maternity care, reduce D&A during childbirth, and improve women’s childbirth experiences. The present study will provide detailed information on a group of Iranian women’s experiences of D&A during childbirth and its related factors to inform the development of the new guideline. The quantitative and qualitative approaches in the mixed method design are integrated to correct possible defects, and provide a better understanding of the subject [38, 39]. The mixed method study will help health professionals understand women’s experiences of D&A during childbirth and prevent any potential harm to these women. Considering the vulnerability of women during pregnancy, particularly during labor, and the fact that D&A can be associated with fear of NVD and increased tendency for performing cesarean sections, service providers should pay more attention to women during childbirth.

Developing a guideline that involves evidence based practice principles, women’s experiences and maternity staff input can provide a new direction to lead other health care professionals, policymakers, and managers with the same characteristic to improve the quality of the care for women across the world and give new families the best start.

## Data Availability

Not applicable.

## Abbreviations

D&A: Disrespect and Abuse
RMC: Respectful Maternity Care
WHO: World Health Organization

## References

[1] World Health Organization (2014). The prevention and elimination of disrespect and abuse during facility-based childbirth.

[2] Diniz SG, de Oliveira SH, de Aguiar AHF, de Carvalho PGC, Carvalho PCA, Aguiar CA (2015). Abuse and disrespect in childbirth care as a public health issue in Brazil: origins, definitions, impacts on maternal health, and proposals for its prevention. J Human Growth Develop.

[3] Ogangah C, Slattery E, Mehta A (2007). Failure to deliver: violations of Women’s human rights in Kenyan health facilities.

[4] Amnesty International (2010). Deadly delivery: the maternal health care crisis in the USA.

[5] Bowser D, Hill K. Exploring Evidence for Disrespect and Abuse in Facility-Based Childbirth: Report of a Landscape Analysis.USAID/TRAction Project; 2010.

[6] Burrowes S, Holcombe SJ, Jara D, Carter D, Smith K (2017). Midwives’ and patients’ perspectives on disrespect and abuse during labor and delivery care in Ethiopia: a qualitative study. BMC Pregnancy Childbirth.

[7] Abuya T, Warren CE, Miller N, Njuki R, Ndwiga C, Maranga A (2015). Exploring the prevalence of disrespect and abuse during childbirth in Kenya. PLoS One.

[8] Okafor II, Ugwu EO, Obi SN (2015). Disrespect and abuse during facility-based childbirth in a low-income country. Int J Gynaecol Obstet.

[9] Freedman LP, Kruk ME (2014). Disrespect and abuse of women in childbirth: challenging the global quality and accountability agendas. Lancet.

[10] Lukasse M, Schroll AM, Karro H, Schei B, Steingrimsdottir T, Van Parys AS (2015). Prevalence of experienced abuse in healthcare and associated obstetric characteristics in six European countries. Acta Obstet Gynecol Scand.

[11] Lukasse M, Vangen S, ØIAN P, Schei B (2011). Fear of childbirth, women's preference for cesarean section and childhood abuse: a longitudinal study. Acta Obstet Gynecol Scand.

[12] Azami-Aghdash S, Ghojazadeh M, Dehdilani N, Mohammadi M (2014). Prevalence and causes of cesarean section in Iran: systematic review and meta-analysis. Iran J Public Health Journal.

[13] Sethi R, Gupta S, Oseni L, Mtimuni A, Rashidi T, Kachale F (2017). The prevalence of disrespect and abuse during facility-based maternity care in Malawi: evidence from direct observations of labor and delivery. Reprod Health.

[14] Kendall-Tackett K (2015). Respectful care during birth= better breastfeeding rates remarkable new statement from WHO calls for the end of disrespect and abuse during childbirth. Clin Lact.

[15] World Health Organization. WHO recommendations: intrapartum care for a positive childbirth experience. Geneva: World Health Organization; 2018. Licence: CC BY-NC-SA 3.0 IGO. 30070803

[16] Swahnberg K, Schei B, Hilden M, Halmesmki E, Sidenius K, Steingrimsdottir T (2007). Patients’ experiences of abuse in health care: a Nordic study on prevalence and associated factors in gynaecological patients. J Psychosom Obstet Gynaecol.

[17] Abuya T, Ndwiga C, Ritter J, Kanya L, Bellows B, Binkin N (2015). The effect of a multi-component intervention on disrespect and abuse during childbirth in Kenya. BMC Pregnancy Childbirth..

[18] Ratcliffe HL, Sando D, Mwanyika-Sando M, Chalamilla G, Langer A, McDonald KP (2016). Applying a participatory approach to the promotion of a culture of respect during childbirth. Reprod Health.

[19] Kujawski SA, Freedman LP, Ramsey K, Mbaruku G, Mbuyita S, Moyo W (2017). Community and health system intervention to reduce disrespect and abuse during childbirth in Tanga region, Tanzania: a comparative before-and-after study. PLoS Med.

[20] Sando D, Ratcliffe H, McDonald K, Spiegelman D, Lyatuu G, Mwanyika-Sando M (2016). The prevalence of disrespect and abuse during facility-based childbirth in urban Tanzania. BMC Pregnancy Childbirth..

[21] Orpin J, Puthussery S, Davidson R, Burden B (2018). Women’s experiences of disrespect and abuse in maternity care facilities in Benue state. Nigeria BMC Pregnancy Childbirth.

[22] Balde MD, Bangoura A, Diallo BA, Sall O, Balde H, Niakate AS (2017). Aqualitative study of women's and health providers' attitudes and acceptability of mistreatment during childbirth in health facilities in Guinea. Reprod Health.

[23] Bohren MA, Vogel JP, Tunçalp Ö, Fawole B, Titiloye MA, Olutayo AO (2016). “By slapping their laps, the patient will know that you truly care for her”: a qualitative study on social norms and acceptability of the mistreatment of women during childbirth in Abuja. Nigeria SSM Popul Health.

[24] Schroll A, Kjrgaard H, Midtgaard J (2013). Encountering abuse in health care;lifetimeexperiences in postnatal women - a qualitative study. BMC Pregnancy Childbirth..

[25] Tashakkori A, Creswell JW (2007). The new era of mixed methods. J Mixed Methods Res.

[26] Manning A, Schaff M. Disrespect and abuse in childbirth and respectful materninty care.2011.availabled from: https://www.whiteribbonalliance.org/wp-content/.../6422_RMC-DA-Brief-Final.pdf.

[27] Kruk MPM, Mbaruku G, de Pinho H, Galea S (2009). Women's preferences for place of delivery in rural Tanza-nia: a population-based discrete choice experiment. Am J Public Health.

[28] Nunnally JC, Bernstein IH. Psychometric theory: 3 rd ed. Mc Graw-hill; 1994.

[29] Sheferaw ED, Bazant E, Gibson H, Fenta HB, Ayalew F, Belay TB (2017). Respectful maternity care in Ethiopian public health facilities. Reprod Health.

[30] Sheferaw ED, Mengesha TZ, Wase SB (2016). Development of a tool to measure women’s perception of respectful maternity care in public health facilities. BMC pregnancy childbirth.

[31] Asefa A, Bekele D (2015). Status of respectful and non-abusive care during facility-based childbirth in a hospital and health centers in Addis Ababa. Ethiopia Reprod Health.

[32] USAID: respectful maternity care standards. USAID; 2011.Availlable from: https://www.k4health.org/sites/default/files/RMC%20Survey%20Report.pdf.

[33] Dencker A, Taft C, Bergqvist L, Lilja H, Berg M (2010). Childbirth experience questionnaire (CEQ): development and evaluation of a multidimensional instrument. BMC Pregnancy Childbirth..

[34] Streubert H, Carpenter D (2010). Qualitative research in nursing: advancing the humanistic imperative.

[35] Hsieh HF, Shannon SE (2005). Three approaches to qualitative content analysis. Qual Health Res.

[36] Graneheim UH, Lundman B (2004). Qualitative content analysis in nursing research:concepts, procedures and measures to achieve trustworthiness. Nurse Educ Today.

[37] Lincoln YS (1995). Emerging criteria for quality in qualitative and interpretive research. Qual Inq.

[38] Polit D, Beck C (2017). Essentials of nursing research.

[39] Creswell J, Clark V (2011). Designing and conducting mixed methods research.

